# Case of Extra-Uterine Conception, Wherein Hystero-Thecotomy Was Successfully Performed

**Published:** 1819-07-01

**Authors:** John King

**Affiliations:** of South Carolina


### *Case of Extra-Uterine Conception, wherein Hystero-thecotomy was successfully performed.* By JOHN KING, Esq. of South Carolina.*

THE subject of this paper had been four days in labour, the pains returning in the usual manner, but without any of the common signs attendant on the natural parturient process. The woman being worn out with unprofitable efforts desired the assistance of Mr. King, who, on examining, *per vaginam*, could not feel the os uteri. Perceiving it to be a case of extra-uterine conception, he determined on the following operation. It consisted simply in laying the vagina open to a great extent. "The head of the fœtus floated and vacillated on the right side of the uterus, and pushed the uterus from its situation. I introduced a small bistoury, guarded by the end of my finger, as far as I possibly could, so as completely to embrace the circumference of the head, and thereby prevent any laceration of the parts in the progress of delivery. I then pierced the vagina through, and carried the knife five or six inches downwards and backwards, so as to insure the easy extrication of the child's head. The instant the vagina was laid open, the waters flowed abundantly, the membranes being laid open with the same incision. I then introduced my hand through the wound in the vagina, and found the infant very high up, and firmly fixed, without any prospect of its descending into the pelvis. I therefore desired the assistants to press gently and constantly upon the abdomen, and to imitate a circular descending motion with their hands." The head was now perceived to descend slowly into the pelvis, and Mr. King was enabled to complete the extraction, with the forceps, after a long and uninterrupted exertion. The child appeared to be still-born, yet Mr. King inflated the lungs through a tube, and "he was pleased to find it had borne the brunt of the day, without a fatal event."

The hæmorrhage from this large incision was inconsiderable, and was probably useful in moderating the subsequent adhesive inflammation. The infant was of the common size, and well conditioned. The placenta was uncommonly small, and the chord remarkably thin.

The morning after delivery the patient was copiously bled, and an anodyne repeated. On interrogation, she declared that she did not know a knife had been used. She was ordered to lie on an inclined plane, with the head very low. On the third day she was

---

* Condensed from "An Analysis of the subject of Extra-Uterine Fœtation, &c." By John King, Esq. one volume, octavo, 176 pages, Callow, Importer, London, 1819.

uncomfortable, with pain over the pubes; and on examination, per vaginam, the intestines were discovered pushing at the wound, which was much contracted. She was directed to lie on the left side, with the hips more elevated, to favour the retraction and gravitation of the intestines from the wound. A blister was applied over the pubes, and a saline anodyne mixture prescribed. The bowels were kept torpid on purpose to avoid purging, until the danger of hernial protrusion was over. In two weeks the woman, tho' without permission, walked about. In two weeks more Mr. King could not perceive any trace of the incision in the vagina; and the uterus had resumed its natural site. "The event of this case, (says Mr. King) is a certain proof that every infant so situated, may stand a great chance of being delivered alive, and no harm whatever can accrue to the mother, cæteris paribus, from the operation."

Norwich, August, 1818.

We are much obliged to the author of the little work, from which the above very interesting case is extracted, for his politeness in transmitting it to us. It contains a series of very ingenious remarks, and many important observations on retroversion of the womb, for which we refer our obstetric brethren to the work itself.

EDIT.

